# Resection of extrahepatic bile ducts with partial hepatectomy for treating intra- and extrahepatic hepatolithiasis

**DOI:** 10.1186/s12893-021-01419-5

**Published:** 2021-12-15

**Authors:** HongTian Xia, XiangFei Meng, XianLei Xin, Tao Yang, Yang Liu, Bin Liang, Jing Wang

**Affiliations:** Faculty of Hepato-Pancreato-Biliary Surgery, Chinese PLA General Hospital, Beijing, 100853 China

**Keywords:** Hepatolithiasis, Hilar bile duct stenosis, Abnormality of the duodenal papilla, Oddis sphincter, Partial hepatectomy

## Abstract

**Background:**

To evaluate the efficacy and safety of our new surgical procedures for primary intra- and extrahepatic hepatolithiasis. Hepatolithiasis is an intractable disease with frequent recurrences.

**Methods:**

From 1996 to 2005, 142 patients with intrahepatic and/or extrahepatic hepatolithiasis treated with the conventional surgical methods were included as the control group, while 128 consecutive patients treated with new surgical methods from 2006 to 2015 were included as the observation group. The new surgical procedures included a comprehensive intraoperative exploration of the bile ducts, focusing on the structure and function of the hilar bile duct and duodenal papilla, exploration of the affected liver, and bile culture.

**Results:**

The observation group had a significantly higher complete stone clearance rate than the control group (100% vs. 65.96%). The observation group had significantly lower incidences of cholangitis and bile duct stones, as well as a higher excellent and good long-term surgical efficacy rate (86.24% vs. 52.73%). Multivariate Cox analysis showed that the control group had a higher risk for fair + poor efficacy than the observation group (HR: 8.47).

**Conclusions:**

Our new surgical procedures are safe and can provide a good long-term efficacy for treating primary hepatolithiasis intra- and extrahepatic hepatolithiasis.

## Introduction

Hepatolithiasis is defined as the presence of stones in the intrahepatic bile ducts, regardless of the coexistence of gallstones in the extrahepatic bile duct [1]. There is a geographic difference in the incidence of hepatolithiasis; the incidence ranges from 30 to 50% in East Asia [2], but is only 0.6–1.3% in Western countries [3]. However, the incidence of hepatolithiasis in Western countries has been increasing as a result of increased immigration from regions with a high incidence [4–6]. Hepatolithiasis is an intractable disease with frequent recurrences [7], and commonly leading to cholangitis, chronic sepsis, and liver abscess, and the need for multiple operative interventions. Moreover, hepatolithiasis is a well-known risk factor for the development of intrahepatic cholangiocarcinoma [8], and a prognostic factor for poor outcomes [9, 10].

Since the 1950 s, Chinese researchers of hepatobiliary surgery led by Professor Huang Zhiqiang have conducted long-term, systematic research on hepatolithiasis [11]. By the end of the 1980 s, theories regarding the pathogenesis of the disease and surgical methods were established. However, the etiology and pathogenesis of the disease were not fully understood [12], and the postoperative recurrence and repeat surgery rates were high. At our institution we have been researching the etiology and pathogenic mechanisms of primary hepatolithiasis for more than 10 years, and with an improved understanding of the disease we have revised surgical techniques for the management of the condition.

In 2006, based on our research of the condition, we established new surgical procedures for primary hepatolithiasis that provides improved and satisfactory outcomes. The purpose of this study is to evaluate efficacy and safety of our new surgical procedures for primary intra- and extrahepatic hepatolithiasis.

## Materials and methods

### Patients

This study was performed in accordance with the Declaration of Helsink and approved by the Institutional Review Board (IRB) of the Chinese PLA General Hospital (Approval No: S2020-392-01).Written informed consent was waived by the IRB due to the retrospective nature of this study.

In 2006, we established systematic diagnostic criteria and new surgical treatment method for intrahepatic and extrahepatic hepatolithiasis. From January 1996 to December 2005, 142 consecutive patients with intrahepatic and/or extrahepatic hepatolithiasis treated with the conventional surgical methods in our hospital were included as the control group. From January 2006 to December 2015, 128 consecutive patients with intrahepatic and/or extrahepatic hepatolithiasis treated with new surgical methods at the Department of Hepatobiliary Surgery of our hospital were included as the observation group.

Inclusion criteria for this analysis were: (1) Definitive diagnosis of primary hepatolithiasis; (2) Received surgical management; (3) Postoperative follow-up duration > 36 months.

Patients with any of the following criteria were excluded: (1) Recurrence of hepatolithiasis after surgery; (2) Secondary hepatolithiasis; (3) Contraindications to laparotomy; (4) End-stage biliary disease with severe portal hypertension and biliary cirrhosis; (5) Cholesterol stones in the intrahepatic and extrahepatic bile duct due to metabolic reasons; (6) Severe congenital anomalies in the bile duct tree; (7) Follow-up duration < 36 months.

The diagnosis of intrahepatic and extrahepatic hepatolithiasis was based on medical history, clinical manifestations, serological examinations, and the results of imaging studies such as abdominal ultrasound, computed tomography (CT), and magnetic resonance imaging (MRI).

### Preoperative evaluation

The surgical plan was determined based on the distribution of bile duct stones, the location of bile duct stenosis, and the extent of lesions in the affected liver as identified on preoperative imaging studies and serological examinations.

Tests of liver function were performed on patients requiring a partial hepatectomy, the liver volume allowing for a safe resection was calculated. The extent of partial hepatectomy was determined according to the distribution of intrahepatic bile duct stones and lesions in the affected liver. The nutritional status of all patients was assessed, and malnourished patients were given appropriate nutritional support.

### Surgical methods for the control group

All patients in the control group received one of 4 surgical procedures.

Surgical method I: Intra- and extrahepatic exploration with stone extraction, T tube drainage.

Surgical method II: Extrahepatic exploration with stone extraction, partial hepatectomy, T tube drainage.

Surgical method III: Intra- and extrahepatic exploration with stone extraction, extrahepatic bile duct resection, Roux-en-Y choledochojejunostomy.

Surgical method IV: Bile duct exploration with stone extraction, extrahepatic bile duct resection, partial hepatectomy, Roux-en-Y choledochojejunostomy.

### Surgical methods for the observation group

**Fig. 1:**
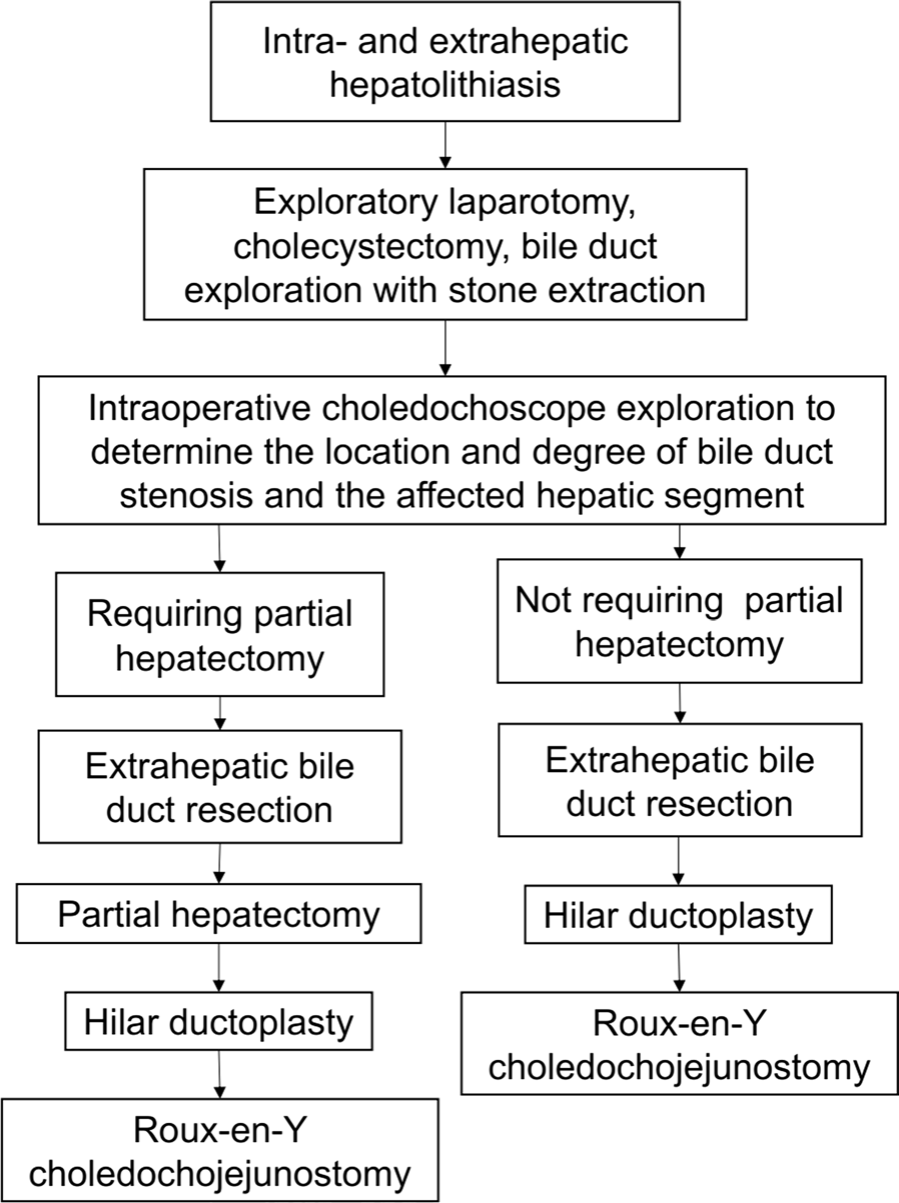
The flowchart of the surgical procedure for the observation group

All patients in the observation group received one of 2 surgical procedures, based on the need for a partial hepatectomy. The flowchart of the surgical procedure for the observation group were shown in Fig. 1.

Surgical method I: Bile duct exploration with stone extraction, intraoperative choledochoscope exploration, extrahepatic bile duct resection, partial hepatectomy, hilar ductoplasty, Roux-en-Y choledochojejunostomy.

Surgical method II: Bile duct exploration with stone extraction, intraoperative choledochoscope exploration, extrahepatic bile duct resection, hilar ductoplasty, Roux-en-Y choledochojejunostomy.

### Bile duct exploration with stone extraction

During surgery, the bile duct was routinely excised to perform bile duct exploration with stone extraction, followed by a comprehensive exploration of the biliary system by intraoperative choledochoscopy. Attention was paid to the structure and function of the hilar bile duct, the lower end of the common bile duct, and the duodenal papillary sphincter to determine the distribution of intra- and extrahepatic bile duct stones, the location of bile duct stenosis, the structure and function of the lower end of the common bile duct and of the duodenal papilla. At the same time, the results of imaging studies were used to determine the degree of lesions in the affected liver, and the need and feasibility of partial hepatectomy were further assessed. Whether or not a partial hepatectomy was performed was determined by considering all of the available data at the time of surgery.

### Intraoperative assessment of the structure and function of the duodenal papilla

There is currently no commonly accepted standard assessment of the structure and function of the duodenal papilla. We assessed the structure and function of the duodenal papilla based on morphological observations, and the choledochoscopic pass-through test. Morphological observations included the diameter of the common bile duct, the inflammation status of the inner wall of the common bile duct, the opening and closing function of the duodenal papilla, and any obvious structural and/or functional abnormalities.

The choledochoscopic pass-through test used 2 choledochoscopes with different diameters. One was a P60 choledochoscope (Olympus, Japan) with an outer diameter = 4.9 mm, and the other was a CD30s ultra-thin choledochoscope (Olympus) with an outer diameter = 2.7 mm. The pass-through tests consisted of determining if the choledochoscopes could successfully pass through the duodenal papilla to the duodenum without any expansion of the papilla. The assessment criteria were: Normal function: The CD30s choledochoscope was able to pass through the duodenal papilla, but the P60 choledochoscope could not. Incomplete closure of the duodenal papilla: The P60 choledochoscope was able to pass through the duodenal papilla. Papillary stenosis: Neither the P60 nor the CD30s choledochoscope was able to pass through the duodenal papilla.

### Hilar ductoplasty

Most cases of intrahepatic and extrahepatic bile duct stones have hilar bile duct stenosis. Therefore, after extrahepatic bile duct resection it is necessary to expand the stricture of the hilar bile duct. At the same time, in order to prevent the complications of hilar duct bile duct stricture following choledochojejunostomy it is also necessary to perform hilar ductoplasty. Hilar ductoplasty was performed as previously described [13]. Briefly, the hilar plate was dissected to expose the junction of the left and right hepatic ducts. The transverse portion of the left hepatic duct and the junction of the right anterior and right posterior hepatic ducts, were further exposed. The transverse portions of the left hepatic duct and the right hepatic duct were dissected along the hepatic duct. When the hilar ductoplasty is completed, the diameter of the hilar bile duct opening should be > 2 cm.

### Exploring the affected liver and partial hepatectomy

Combined with preoperative imaging data, intraoperative exploration of the affected liver was performed to determine if liver atrophy/fibrosis was present, and the extent. Based on examination of the liver and the results of bile duct exploration, it was determined whether the affected liver should be excised.

Partial hepatectomy is to remove the diseased bile duct and the affected hepatic segment at the same time. Partial hepatic segment resection was performed based on the distribution of diseased bile ducts. The purpose of partial hepatectomy is to ensure the radical treatment of hepatolithiasis, and to avoid the unnecessary expansion of the scope of the surgery. If bile duct stenosis is identified at the first- or second-order bile duct near the hepatic hilum after removing the stones, the bile duct obstruction can be relieved by hilar ductoplasty and there is no need for partial hepatectomy of the affected liver. If the bile duct stenosis is located at the opening of a secondary- or tertiary-order bile duct in the liver, it cannot be relieved by hilar ductoplasty. In this situation, a partial hepatectomy should be performed to achieve proper postoperative bile duct flow of the intrahepatic bile duct.

Another important factor for determining the need for partial hepatectomy is the pathological degree of lesions in the affected liver. If the affected liver has obvious fibrosis, ischemic atrophy of liver tissue, or biliary cirrhosis normal function of the affected portion of the liver is unlikely to be restored. Thus, a partial hepatectomy should be performed. On the other hand, when the affected liver had normal circulation and is not atrophic normal function can be restored by removing the stones and eliminating the bile duct stenosis. In this situation, the affected liver should be retained and a partial hepatectomy is not required.

In some patients, the intrahepatic bile duct stones are diffusely distributed, and stenosis of the hilar bile duct is so severe that it cannot be treated by hilar ductoplasty and the stones in the intrahepatic bile duct cannot be easily removed. In this situation, a partial hepatectomy should be considered to remove the relatively severely diseased liver tissue and the severely narrowed hilar bile duct. After the partial hepatectomy, it is relatively easy to remove the stones in the remaining liver and bile ducts, and to establish proper postoperatively intrahepatic bile duct flow.

### Indications and contraindications for partial hepatectomy

#### Indications

(1) The distribution of intrahepatic bile duct stones is limited to a certain liver segment or lobe, and the intrahepatic lesions can be completely removed by partial hepatectomy. (2) The structure and function of the affected liver are damaged, and will not recover after removal of the stone. (3) The intrahepatic bile duct stricture site is at a relatively high level, hilar ductoplasty will not achieve proper bile duct flow in the intrahepatic bile duct. (4) Sufficient liver volume will remain after partial hepatectomy such that liver function will be normal. (5) Intrahepatic bile duct stones are diffusely distributed. In this situation, a partial hepatectomy will not completely remove all intrahepatic lesions, but will eliminate the hepatic hilar bile duct stenosis to ensure proper bile duct flow in the remaining intrahepatic bile ducts. The primary purpose of the partial hepatectomy in this case is to establish the proper bile duct flow in the intrahepatic bile duct, so as to reduce the occurrence of postoperative complications and to achieve a good long-term outcome.

#### Contraindications

(1) A large portion of the liver is affected, and the liver function will be inadequate after removal of all of the diseased liver. (2) Biliary cirrhosis and decompensated liver function. (3) A partial hepatectomy will not achieve proper bile duct flow in the intrahepatic bile duct if the intrahepatic bile duct stones are widely distributed, the stricture site of the intrahepatic bile duct is at a relatively high level, or narrow bile ducts are widely distributed.

### The Roux-en-Y choledochojejunostomy

After stone removal and thorough exploration of the biliary system, the common bile duct was radically excised as much as possible to avoid complications after the biliary-enteric anastomosis. When resecting the lower end of the common bile duct, special care should be taken not to create a dead space, thus avoiding postoperative infection, cholangitis, and pancreatitis.

The purpose of the extrahepatic bile duct resection is to address the problem of hilar bile duct stenosis. Therefore, after extrahepatic bile duct resection the hilar ductoplasty was performed to ensure a sufficient size of the opening of biliary-enteric anastomosis, and to avoid postoperative complications. Finally, the Roux-en-Y choledochojejunostomy was performed.

### Short-term efficacy assessment

The short-term efficacy was assessed during hospitalization, and included the successful operation rate, the rate of stone clearance, the surgery duration, the amount of intraoperative blood loss, the amount of blood transfusion, and postoperative hospital length of stay. Postoperative complications during hospitalization were recorded, and included hemorrhage, bile leakage, pancreatic leakage, abdominal infection, and delayed incision healing.

### Long-term efficacy assessment

All patients were followed-up at out hospital or their local hospital. Patients seen at local hospitals with problems or abnormalities were referred to our hospital for evaluation. Patients were seen every 3 months for the first year after surgery, then every 6 months for years 2–5 after surgery, and then yearly. Patients with complications were seen as necessary.

During follow-up, postoperative biliary function was evaluated by consideration of symptoms such as abdominal pain, fever, and jaundice. Blood biochemical examinations performed at follow-up visits included measurement of serum alanine aminotransferase (ALT), aspartate aminotransferase (AST), alkaline phosphatase (ALP), and gamma-glutamyltransferase (γ-GT) levels. Abdominal ultrasound, CT, MRI were performed to identify postoperative biliary-enteric anastomotic stricture, recurrence of common bile duct stones (CBDS), and postoperative malignant transformation.

Assessment of long-term biliary function was based on the Mayo clinic score [14], and the postoperative assessment system of Lillemoe et al. [15], with modification to include evaluation of the formation of cystic dilation of the bile ducts, as previously described [16]. In brief, biliary function was scored as: *excellent*, biochemical indicators were normal and there were no clinical symptoms or anatomical abnormalities; *good*, no clinical manifestations of cholangitis, only a few small bile ducts exhibiting an abnormal structure, biochemical indicators were normal, and no medical intervention was necessary; *fair*, mild anatomical abnormalities of bile ducts were present, and clinical manifestations of cholangitis were observed < 3 times/year and the episodes were relieved by conservative treatments such as antibiotics; *poor*, repeated episodes of cholangitis, anatomical structure of the bile ducts was abnormal, bile duct strictures and stones were present, and reoperation was required.

Postoperative complications were graded according to the Clavien-Dindo Classification [17].

### Statistical analysis

Continuous variables were reported with mean ± standard deviation (SD) and were compared using Student’s independent t-test or Mann-Whitney U test (if normality was not assumed). Categorical variables were presented as number and percentage and were compared using Chi-square test or Fisher’s exact text (if expected value ≤ 5 was found). Survival analyses, including Kaplan-Meier survival test and Cox proportional-hazards model, were used to investigate the difference of long-term efficacy in follow-up period where patient’s efficacy was integrated into dichotomous outcomes: excellent + good vs. fair + poor (as event). All analyses were done using IBM SPSS Version 25 (SPSS Statistics V25, IBM Corporation, Somers, New York). The statistical significance level for all the tests was set at a P-value < 0.05, two-tailed.

## Results

### Patient characteristics

A total of 270 patients, 116 males and 154 females, with a mean of 45.83 ± 12.27 years (range: 22–78 years) with hepatolithiasis treated at our institution were included in this study. There were 142 and 128 patients in the control and observation groups, respectively. Of the 270 patients, bile duct stones were limited in distribution in 201 patients (74.44%) while 69 patients (25.56%) had diffusely distributed stones. Except for imaging diagnosis and surgical methods, there was no significant difference in patients’ demographic and clinical characteristics, including age, gender, γ-GT, ALP, and stone distribution between two groups (Table 1, all P > 0.05), indicating the comparativeness between control and observation groups.

**Table 1:** Demographic and clinical characteristics

Parameters	Control (n = 142)	Observation (n = 128)	All (n = 270)	P
Age, year	45.73 ± 12.02	45.94 ± 12.60	45.83 ± 12.27	0.897
Gender				0.999
Male	61 (42.96%)	55 (42.97%)	116 (42.96%)	
Female	81 (57.04%)	73 (57.03%)	154 (57.04%)	
Imaging diagnosis method				0.009
Ultrasound, CT, MRCP	114 (80.28%)	116 (90.63%)	230 (85.19%)	
Ultrasound, MRCP	8 (5.63%)	7 (5.47%)	15 (5.56%)	
CT, MRCP	9 (6.34%)	5 (3.91%)	14 (5.19%)	
Ultrasound, CT	11 (7.75%)	0 (0.00%)	11 (4.07%)	
γ-Glutamyltransferase				0.895
Elevation	136 (95.77%)	123 (96.09%)	259 (95.93%)	
Normal	6 (4.23%)	5 (3.91%)	11 (4.07%)	
Alkaline phosphatase				0.917
Elevation	140 (98.59%)	126 (98.44%)	266 (98.52%)	
Normal	2 (1.41%)	2 (1.56%)	4 (1.48%)	
Stone distribution				0.842
Limited distribution	105 (73.94%)	96 (75.00%)	201 (74.44%)	
Diffuse stone	37 (26.06%)	32 (25.00%)	69 (25.56%)	
Surgical methods				< 0.001
Surgical Methods I	24 (16.90%)	93 (72.66%)	117 (43.33%)	
Surgical Methods II	22 (15.49%)	35 (27.34%)	57 (21.11%)	
Surgical Methods III	67 (47.18%)	0 (0.00%)	67 (24.81%)	
Surgical Methods IV	29 (20.42%)	0 (0.00%)	29 (10.74%)	

ERCP/EST, Endoscopic retrograde cholangiopan- creatography/endoscopic sphincterotomy; *CBDE, Common bile duct exploration *; LC+LCBDE, Laparoscopic cholecystectomy plus laparoscopic common bile duct exploration; *LC, laparoscopic cholecystectomy *; CT, computed tomography

Surgical method I: exploratory laparotomy, bile duct exploration with stone extraction, extrahepatic bile duct resection, partial hepatectomy, hilar ductoplasty, Roux-en-Y choledochojejunostomy

Surgical method II: exploratory laparotomy, bile duct exploration with stone extraction, extrahepatic bile duct resection, hilar ductoplasty, Roux-en-Y choledochojejunostomy

Surgical method III: exploratory laparotomy, bile duct exploration with stone extraction, partial hepatectomy, T-tube drainage

Surgical method IV: exploratory laparotomy, bile duct exploration with stone extraction, T-tube drainage

### Choice of surgical methods

**Fig. 2:**
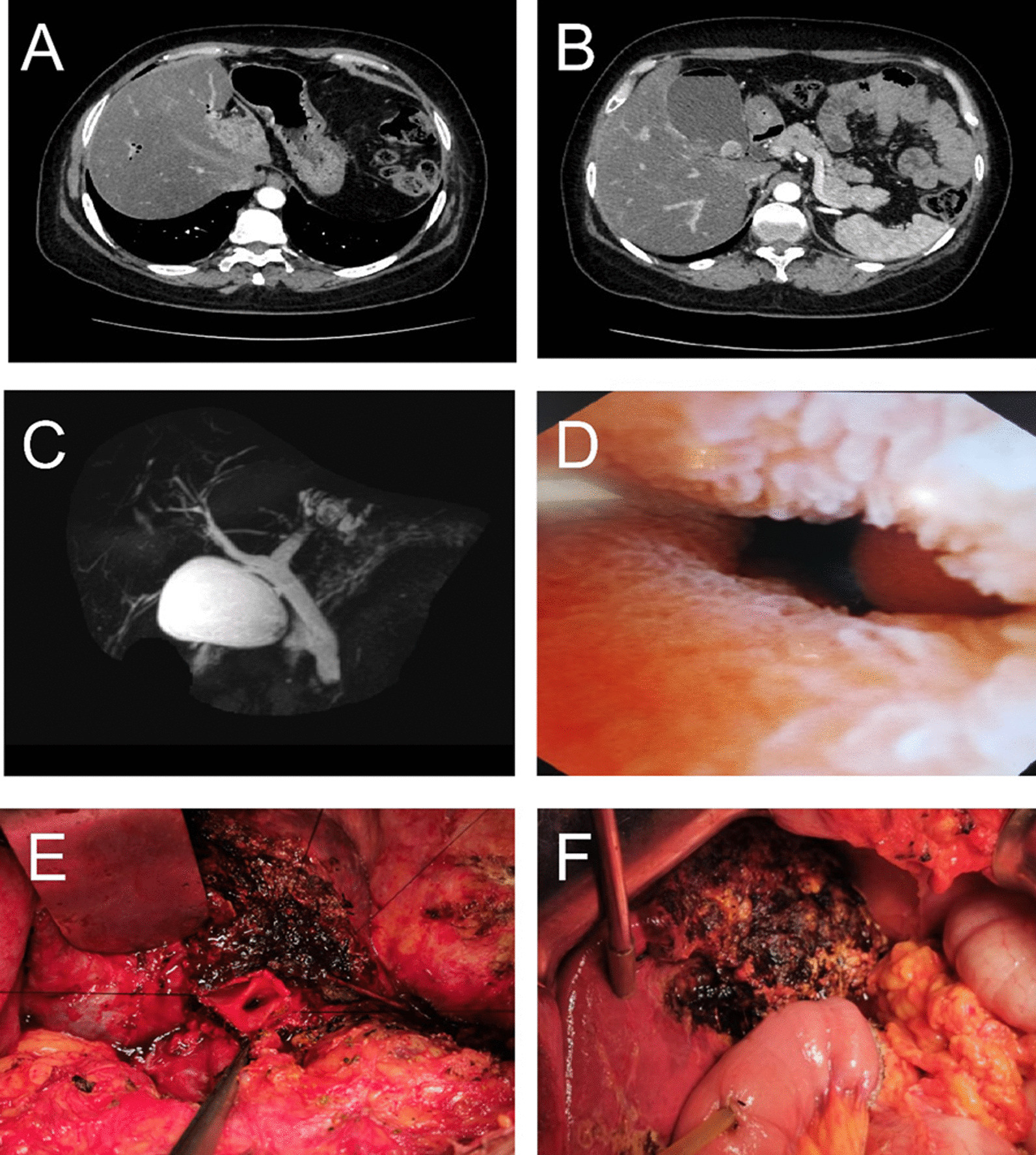
A representative case in the observation group (female, 51 years old). The CT images showed intrahepatic bile duct stones (**A**) and common bile duct stones (**B**). **C** MRCP image displayed left intrahepatic bile duct stones, left hepatic duct stenosis, and common bile duct stones. **D** Intraoperative choledochoscope exploration showed incomplete closure of the duodenal papilla at the lower end of the common bile duct. The patient underwent left hepatectomy, hilar ductoplasty (**E**) and Roux-en-Y choledochojejunostomy (**F**)

In the observation group, 93 patients (72.7%) received a partial hepatectomy (surgical method I), including left lateral lobectomy (n = 26, 27.96%), left hepatectomy (n = 31, 33.33%), and right posterior lobectomy (n = 22, 23.66%), and right hepatectomy (n = 14, 15.05%). The remaining 35 patients (27.3%) were treated with surgical method II (without a partial hepatectomy). The photographs of a representative case in the observation group were shown in Fig. 2.

In the control group, 24 patients (16.90%) and 22 patients (15.49%) were treated with the surgical method I and II. There were 67 patients (47.18%) in the control group were treated with surgical method III ( bile duct exploration with stone extraction, partial hepatectomy, T-tube drainage), the other 29 patients (20.42%) were with surgical method IV (surgical method III without a partial hepatectomy).

### Intraoperative findings and surgical outcomes of the observation group

Intraoperative choledochoscopic bile duct exploration showed that a large proportion of patients had structural abnormalities in the duodenal papilla. As shown in Tables 2 and 96 patients (75.0%) had a clear structural abnormality at the lower end of the common bile duct, including 35 patients (27.3%) with stenosis at lower common bile duct and 61 patients (47.7%) with incomplete closure of lower end of the common bile duct. In the remaining 32 patients (25.0%), intraoperative choledochoscopic bile duct exploration revealed no structural abnormalities at the lower end of the common bile duct. However, it should be noted that a finding of no structural abnormalities does not rule out functional abnormalities in the lower end of the common bile duct. Currently, there is no other method to accurately assess function.

**Table 2:** Surgical parameters and perioperative outcomes

Parameters	Control (n = 142)	Observation (n = 128)	All (n = 270)	P
Choledochoscopic exploration finding at the lower end of the bile duct				–
Stricture	–	35 (27.34%)	(0.00%)	–
Sphincter of Oddi insufficiency	–	61 (47.66%)	(0.00%)	–
No abnormal finding	–	32 (25.00%)	(0.00%)	–
Choledochoscopic exploration at hilar bile duct				–
Stricture	–	122 (95.31%)	(0.00%)	–
No abnormal finding	–	6 (4.69%)	(0.00%)	–
Intraoperative bile bacterial culture				0.635
Negative	17 (11.97%)	13 (10.16%)	30 (11.11%)	
Positive	125 (88.03%)	115 (89.84%)	240 (88.89%)	
Complete stone clearance				< 0.001
Yes	93 (65.96%)	128 (100.00%)	221 (82.16%)	
No	48 (34.04%)	0 (0.00%)	48 (17.84%)	
Post-operative hospitalization, day	10.30 ± 2.58	10.24 ± 2.76	10.27 ± 2.66	0.852
Short-term postoperative complications				
Pure bile leakage	1 (0.70%)	5 (3.91%)	6 (2.22%)	0.105
Bile leakage with abdominal infection	6 (4.23%)	5 (3.91%)	11 (4.07%)	0.895
Incision delayed healing	8 (5.63%)	6 (4.69%)	14 (5.19%)	0.726
Postoperative abdominal hemorrhage	2 (1.41%)	2 (1.56%)	4 (1.48%)	1.000

Choledochoscopic bile duct exploration showed that the majority of patients (122, 95.3%) had hilar bile duct stenosis (Table 2). Here, hilar bile duct stenosis includes absolute and relative stenosis, and hilar bile ducts refers to the common hepatic duct and the left and right hepatic ducts, as well as the right anterior and posterior right hepatic ducts, and the part of the bile duct in the hepatic tail lobe that open at the hilar area. This result indicates that hilar bile duct stenosis is common in patients with intra- and extrahepatic bile duct stones, and is an important cause of intrahepatic bile duct stones.

The mean operation duration was 173.7 ± 44.3 min (range: 120–300 min). The longest operation duration was 300 min, which was due to a wide distribution of intrahepatic bile duct stones, and stone removal and right posterior sectionectomy were time-consuming. Only 5 patients required an intraoperative blood transfusion (range: 200–600 mL).

### Comparison of intraoperative findings and surgical outcomes between two groups

As shown in Table 2, intraoperative bile cultures were positive in 128 (88.03%) and 115 (89.84%) patients of control and observation groups, respectively (P = 0.635). The rate of complete stone clearance was significantly higher in the observation group than in the control group (100% vs. 65.96%, P < 0.001). The post-operative hospitalization stay of control and observation groups were 10.30 ± 2.58 and 10.24 ± 2.76 days (P = 0.852).

### Short-term therapeutic efficacy

In observation group, the operation was successfully completed in all the 128 patients, with a success rate of 100%. Use of intraoperative choledochoscopy resulted in an intraoperative stone removal rate of 100%. All patients reported relief of symptoms after surgery, and all patients were discharged in good condition, there were no deaths related to the surgery.

The mean postoperative hospital stay of control and observation groups were 10.30 ± 2.58 days (range: 7–21 days) and 10.24 ± 2.76 days (range: 7**–**21 days) (P = 0.852). As indicated in Table 2, during hospitalization, 25 patients (9.26%) had postoperative complications: 17 cases of bile leakage (6 cases of pure bile leakage, 11 cases of bile leakage with abdominal infection), 14 cases of delayed wound healing, and 4 cases of postoperative abdominal hemorrhage. No significant difference was found in the incidence of short-term postoperative complications between groups (all P > 0.05). Bile leakages were resolved by drainage, and abdominal infections were resolved by treatment with antibiotics and peritoneal irrigation and drainage. Delayed wound healing was resolved by wound care with frequent dressing changes. The patients with postoperative abdominal hemorrhage were treated with transhepatic arterial embolization.

### Follow-up and long-term therapeutic efficacy

Of the 270 patients, 221 patients (81.85%) achieved long-term follow-up (Table 3). Forty-nine patients were lost to follow-up because they did not keep scheduled follow-up appointments and/or they had moved and could not be contacted. The mean follow-up time was 99.67 ± 29.87 months (range: 36–156 months), which was not significantly different between the two groups (P = 0.229).

**Table 3:** Follow-up and long-term complications

Parameters	Control (n = 142)	Observation (n = 128)	All (n = 270)	P
Follow-up				0.181
Yes	112 (78.87%)	109 (85.16%)	221 (81.85%)	
No	30 (21.13%)	19 (14.84%)	49 (18.15%)	
Follow-up period, month	97.54 ± 25.42	101.87 ± 33.81	99.67 ± 29.87	0.229
Long-term complications				
Upper abdominal discomfort	15 (13.64%)	19 (17.43%)	34 (15.53%)	0.438
Bloating	14 (12.73%)	17 (15.60%)	31 (14.16%)	0.543
Cholangitis	36 (32.73%)	9 (8.26%)	45 (20.55%)	< 0.001
Occasional cholangitis	15 (13.64%)	5 (4.59%)	20 (9.13%)	0.020
More severe cholangitis	1 (0.91%)	1 (0.92%)	2 (0.91%)	1.000
Bile duct stones	36 (32.73%)	6 (5.50%)	42 (19.18%)	< 0.001
Cholangiocarcinoma	3 (2.73%)	2 (1.83%)	5 (2.28%)	1.000
Stricture	1 (0.91%)	2 (1.83%)	3 (1.37%)	0.622
Diarrhoea	0 (0.00%)	1 (0.92%)	1 (0.46%)	0.498

During follow-up, 65 patients (29.68%) developed recurrent cholangitis, and in 20 the condition was occasional transient cholangitis which was treated with antibiotics and symptomatic treatment (Table 3). The other 2 patients had more severe symptoms and more frequent attacks. All the 2 patients had recurrent biliary duct stenosis or bile duct stones, and recovered well after reoperation. The incidences of overall cholangitis and bile duct stones were significantly higher in the control group than in the observation group (all P < 0.05). As shown in Table 3, no significance was found in other long-term complications between the two groups, including upper abdominal discomfort, bloating, cholangiocarcinoma, stricture, and diarrhea (all P > 0.05).

Based on the grading system used to measure long-term therapeutic efficacy, 118 patients achieved excellent outcomes (53.88%), 34 (15.53%) good outcomes, 25 (11.42%) fair outcomes, and 42 patients (19.18%) had a poor outcome (Table 4). The excellent and good long-term surgical efficacy rate was significantly higher in the observation group than in the control groups (86.24% vs. 52.73%, P < 0.001).

**Table 4:** Long-term therapeutic efficacy

Parameters	Control (n = 142) (%)	Observation (n = 128) (%)	All (n = 270) (%)	P
Excellent	43 (39.09)	75 (68.81)	118 (53.88)	< 0.001
Good	15 (13.64)	19 (17.43)	34 (15.53)	
Fair	16 (14.55)	9 (8.26)	25 (11.42)	
Poor	36 (32.73)	6 (5.50)	42 (19.18)	

**Fig. 3:**
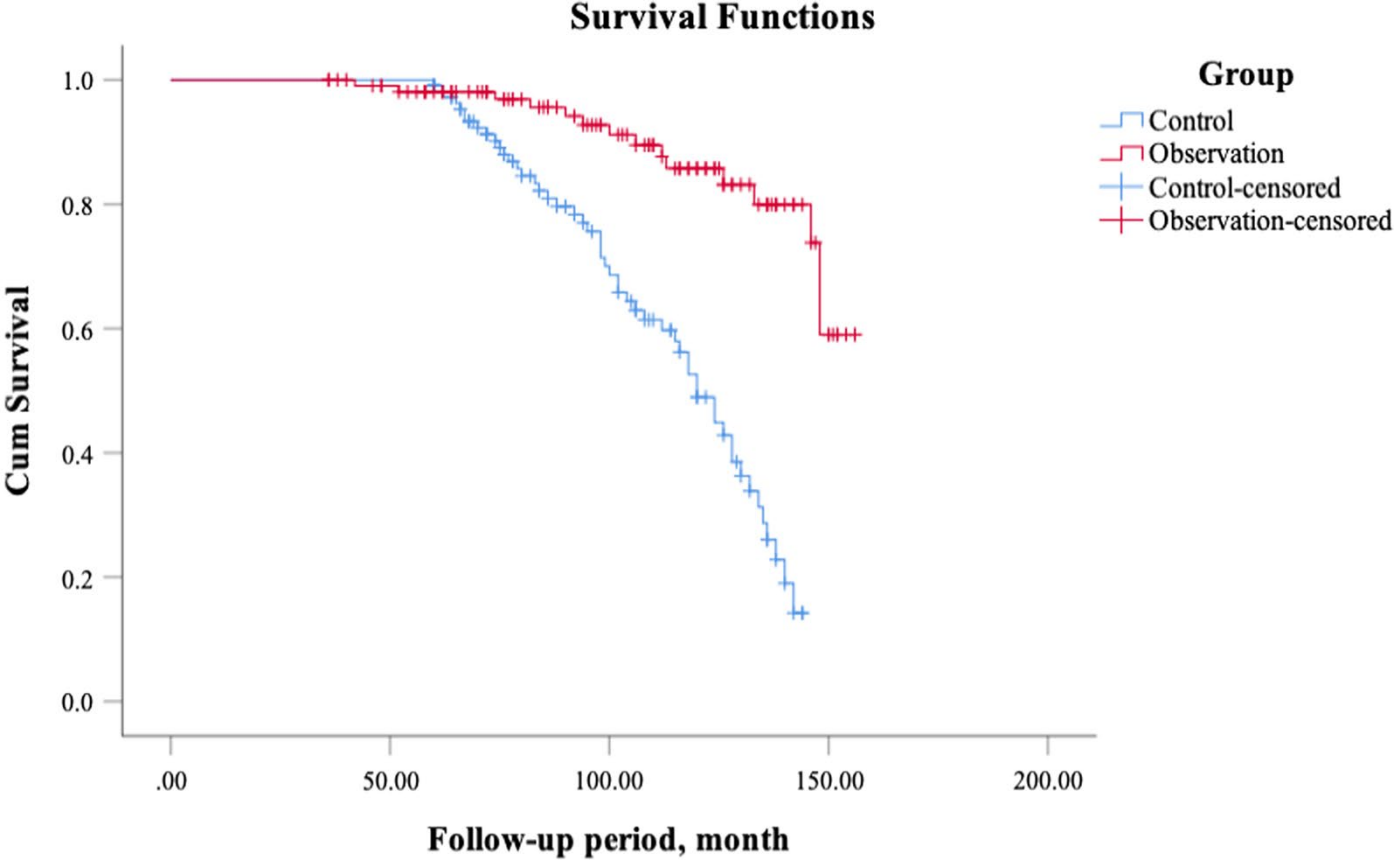
Kaplan-Meier survival function between control and observation groups, the dichotomous outcomes were excellent+good vs. fair+poor (as event). The comparison test was log-rank test, P < 0.001

To further confirm the difference of long-term efficacy in follow-up, survival analysis was performed. As shown in Fig. 3, the long-term trend of excellent and good results was significantly higher in the observation group than in the control group (log-rank test, P < 0.001). In a multivariate Cox proportional-hazards model adjusting for imaging diagnosis and surgical methods, the control group had a significantly higher risk for fair + poor efficacy as compared with the observation group (HR: 8.47, 95% CI 3.91 to 18.36; P < 0.001). These results indicated that the observation group had better long-term efficacy than the control group.

## Discussion

Although the etiology and pathogenesis of primary hepatolithiasis have not been fully elucidated, biliary infection and cholestasis are considered to be necessary for the development of hepatolithiasis [18, 19]. Bacterial infection is an important factor in the formation of pigment stones in intrahepatic bile ducts [20]. Biliary infections include bacterial infections and parasitic infections [21]. However, due to improvement of sanitation the incidence of biliary ascariasis has decreased significantly. In this study, approximately 90% of patients had positive bile cultures. The bacterial species in the bile cultures were mainly Gram-negative bacteria and anaerobic bacteria, which is consistent with intestinal bacteria [22].

Biliary infection is considered to have 2 possible sources, descending infection and ascending infection due to reflux. In descending infection, bacteriobilia is believed to occur via the portal venous system, while in ascending infection intestinal bacteria enter the biliary tract through the duodenal papilla [23–25]. Oddis sphincter dysfunction allows bacteria-containing intestinal fluid to reflux into the biliary tract [26]. In the current study, we observed that 75.0% of patients had a structural abnormality at the lower end of the common bile duct. In addition, the incidence of structural and functional abnormalities of the Oddis sphincter in China is relatively high [27], which is consistent with the high incidence of primary hepatolithiasis in China [28]. These observations suggest that ascending infection is the main cause of bacterial bile infection in primary hepatolithiasis.

Cholestasis is another necessary condition for the formation of primary bile duct stones [29]. Our intraoperative choledochoscopic bile duct exploration showed that in the case of hepatolithiasis bile duct stenosis was mainly present at the lower end of the bile duct and the hilar bile duct. Structural and functional abnormalities of the Oddis sphincter in the lower bile duct can result in cholestasis as well as bacterial infection [18, 19]. In addition to finding that 75.0% of patients had a structural abnormality of the Oddis sphincter at the lower end of the common bile duct, we found that 95.3% of patients had hilar bile duct stenosis. These results suggested that Oddis sphincter structural abnormalities in the lower bile ducts, and hilar bile duct strictures, are common in hepatolithiasis, indicating that structural and functional abnormalities of the bile duct system are an important etiology of the formation of hepatolithiasis. It is now clear that Oddis sphincter dysfunction allows intestinal bacteria in the duodenum to enter the bile duct system and multiply in the bile duct segment with cholestasis, leading to biliary infection. Repeated infections cause bile duct stenosis, in turn aggravating cholestasis. Cholestasis and bacterial infections cause the formation of bile duct stones, which further aggravates bile duct infections. This continuous, vicious cycle gradually causes disease progression.

Based on our research and surgical experience, we have established basic principles for the surgical treatment of primary hepatolithiasis: eliminating lesions, controlling infection, eliminating obstruction, and restoring proper bile duct flow. Eliminating lesions is the basis for treating primary hepatolithiasis, and there are 2 aspects to this principle. One is stone removal. Stones need to be removed as much as possible during the operation. The other is to ensure proper postoperative functions of the liver and biliary system. Liver tissue and bile ducts which have lost normal function to the degree that recovery is not likely should be excised. Therefore, a partial hepatectomy may be required. Controlling infection is an important part of the treatment of hepatolithiasis. Because structural and functional abnormalities of duodenal papillae cannot be repaired, choledochojejunostomy is the only treatment option to block bacterial infection [26]. Choledochojejunostomy can prevent intestinal bacteria from entering the biliary tract, and resolve cholestasis due to structural abnormalities in the Oddis sphincter. Eliminating obstruction is the surgical removal of bile duct stenosis is an important part of the treatment for primary hepatolithiasis, and is crucial for achieving good long-term outcomes and preventing recurrent hepatolithiasis. In addition, choledochojejunostomy cannot completely block intestinal bacteria from entering the biliary tract, so removing bile duct stenosis to eliminate biliary obstruction is important. Because most cases of primary bile duct stones have hilar bile duct stenosis, it is necessary to perform hilar ductoplasty to relieve hilar bile duct stenosis. Proper bile duct flow refers to establishing normal bile flow in the biliary tract [30], and thereby restoring the physiological function of the biliary system. This is the fundamental purpose of the surgical management of hepatolithiasis.

Compared to surgical method IV for the control group, the surgical methods for the observation group used intraoperative choledochoscope to perform detailed exploration of the intra- and extra-hepatic bile ducts to more accurately determine the location and degree of bile duct stenosis. Meanwhile, the structure and function of the duodenal papilla at the lower end of the common bile duct were also more accurately evaluated. These findings can provide a reference for the choice of surgical methods (such as the scope of partial hepatectomy) and biliary-enteric anastomosis to improve surgical effectiveness. Of the 128 patients of the observation group, approximately 73% received a partial hepatectomy (surgical method I). Of the 96 patients with a limited distribution of intrahepatic bile duct stones, 80% received a partial hepatectomy, while of the 32 patients with a diffuse stone distribution 50% received a partial hepatectomy. This result indicates that to achieve good long-term outcomes, the majority of patients with hepatolithiasis will require a partial hepatectomy.

A major limitation of Roux-en-y choledochojejunostomy is that it destroys the anti-reflux function of the duodenal papilla, which may result in reflux cholangitis and subsequent severe complications such as biliary-enteric anastomosis stenosis and hilar bile duct stenosis [13]. Post-choledochojejunostomy biliary-enteric anastomosis stenosis is mainly caused by ischemia and hypoxia that can occur in the bile duct wall at the biliary-enteric anastomotic site [13]. During choledochojejunostomy, the blood vessels supplying the bile duct are severed, resulting in ischemia of the bile duct tissue [26]. When a biliary-enteric anastomosis is performed on bile duct tissue that has undergone ischemia/hypoxia, tissue contracture, fibrous tissue hyperplasia, and scar formation can occur in the bile duct wall tissue, eventually resulting in biliary-enteric anastomotic stenosis [31].

Post-choledochojejunostomy hilar bile duct stenosis is caused by the unique anatomical structures of the hilar bile duct and the hilar plate [13]. There are abundant smooth muscle tissue and fibrous connective tissue in the hilar bile duct and its surrounding hilar plate, which can become inflamed or infected when there is reflux of intestinal fluid after a choledochojejunostomy [30]. Scar formation can be attributed to the proliferation of the smooth muscle tissue and fibrous connective tissue of the hilar bile duct and its surrounding hilar plate. A large amount of fibrous connective tissue hyperplasia eventually leads to hilar bile duct stenosis [32]. The bile duct stenosis leads to intrahepatic bile duct cholestasis, which further aggravates the biliary infection, and makes the post-choledochojejunostomy complications worse [30].

Based on the abovementioned analysis of post-choledochojejunostomy complications, we modified the surgical procedure in an attempt to reduce complications. To address ischemia of the extrahepatic bile duct, a high-site resection of the extrahepatic bile duct was performed by extending the resection range to the hilar bile duct. Because the intrahepatic and the extrahepatic bile ducts have separate blood supply systems, resection of the extrahepatic bile duct fundamentally solves the problem of ischemic bile duct tissue. To prevent biliary-enteric anastomotic stenosis, we adopted hilar ductoplasty as part of the surgical procedure for primary hepatolithiasis. Hilar ductoplasty not only expands the diameter of biliary-enteric anastomosis, but also eliminates the impact of fibrous connective tissue of the hilar plate on the biliary-enteric anastomosis. Hilar ductoplasty ensures proper postoperative bile duct flow of the intrahepatic bile duct and significantly reduces the incidence of postoperative recurrence of cholangitis and biliary-intestinal anastomosis stenosis. Moreover, the hilar ductoplasty can reduce unnecessary hepatectomy and preserve the function of the liver. In addition, as described previously we modified the Roux-en-Y choledochojejunostomy method. Comparison between two groups showed that the observation group receiving the new surgical procedures can achieve a higher stone clearance rate, lower incidences of long-term complications (cholangitis and bile duct stones), and a higher excellent and good long-term surgical efficacy rate.

## Conclusion

Primary hepatolithiasis is an infectious biliary tract disease. Bacterial infection of the biliary tract is the main cause of hepatolithiasis, while structural and functional abnormalities of the hilar bile ducts and duodenal papilla (Qddis sphincter) are necessary for the development of the condition. Our new surgical procedures are safe and can provide a good long-term efficacy for treating primary hepatolithiasis intra- and extrahepatic hepatolithiasis.

## Data Availability

The datasets generated during and/or analysed during the current study are available from the corresponding author on reasonable request.
